# Successful treatment of post chemotherapy esophageal cicatricial atresia in a pediatric patient with anaplastic large cell lymphoma through minimally invasive esophagectomy: a case report

**DOI:** 10.1186/s40792-021-01108-8

**Published:** 2021-02-05

**Authors:** Yuto Hozaka, Ken Sasaki, Takuro Nishikawa, Shun Onishi, Masahiro Noda, Yusuke Tsuruda, Yasuto Uchikado, Yoshiaki Kita, Takaaki Arigami, Shinichiro Mori, Kosei Maemura, Satoshi Ieiri, Yoshifumi Kawano, Shoji Natsugoe, Takao Ohtsuka

**Affiliations:** 1 Department of Digestive Surgery, Breast and Thyroid Surgery, Graduate School of Medical and Dental Sciences, Kagoshima University, 8-35-1 Sakuragaoka, Kagoshima-shi, Kagoshima, 890-8520 Japan; 2 Department of Pediatrics, Graduate School of Medical and Dental Sciences, Kagoshima University, 8-35-1 Sakuragaoka, Kagoshima-shi, Kagoshima, 890-8520 Japan; 3 Department of Pediatric Surgery, Graduate School of Medical and Dental Sciences, Kagoshima University, 8-35-1 Sakuragaoka, Kagoshima-shi, Kagoshima, 890-8520 Japan

**Keywords:** Esophageal stricture, Esophageal atresia, Esophageal obstruction, Esophagectomy, Anaplastic large cell lymphoma, Anaplastic lymphoma kinase, Allogeneic hematopoietic stem cell transplantation, Allogeneic bone marrow transplantation

## Abstract

**Background:**

Anaplastic large cell lymphoma (ALCL) is a CD30-positive T-cell lymphoma, which is a rare type of non-Hodgkin lymphoma. ALCL rarely presents in the gastrointestinal tract, and the esophageal involvement in of ALCL is extremely rare.

**Case presentation:**

An 11-year-old boy who complained of abdominal pain and cough was diagnosed with ALK-positive ALCL on the basis of systemic lymphadenopathy findings and immunohistochemistry results of pleural effusion. Although remission was observed after chemotherapy at 5 months after diagnosis, dysphagia persisted, and esophagoscopy revealed a severe stricture in the middle thoracic esophagus. At 9 months after diagnosis, allogeneic bone marrow transplantation was performed to ensure that complete remission was maintained; however, dysphagia and saliva retention did not improve. Approximately 10 months after diagnosis, esophagoscopy revealed a blind end in the middle thoracic esophagus, similar to that in congenital esophageal atresia. Subsequently, we performed minimally invasive subtotal esophagectomy under thoracoscopy and laparoscopy and gastric conduit reconstruction via the retrosternal route more than 2 years after allogeneic bone marrow transplantation. The final pathological diagnosis was esophageal atresia with esophagitis, with no malignancy. During postoperative evaluation, the patient required swallowing training for a few months, although no major complications were noted. Oral intake was possible, and complete remission was maintained at 14 month post-surgery.

**Conclusions:**

Oncologists must consider the possibility of acquired esophageal cicatricial atresia as a complication during chemotherapy for ALCL. If esophageal obstruction or esophageal atresia occur and if remission is maintained, esophagectomy and esophageal reconstruction are useful treatment options for maintaining oral intake.

## Background

Anaplastic large cell lymphoma (ALCL) is a rare CD30-positive T-cell non-Hodgkin lymphoma. The 2017 World Health Organization classification, which is the most recent classification, categorizes ALCL into anaplastic lymphoma kinase (ALK)-positive ALCL and ALK-negative ALCL [1]. The extranodal involvement of ALCL is rarer in the gastrointestinal tract than in the bones and bone marrow [2, 3]. Esophageal involvement of ALCL is even more rare [4]. Herein, we report the first case of acquired esophageal cicatricial atresia that was successfully treated with minimally invasive esophagectomy in a child with ALK-positive ALCL that showed complete remission after chemotherapy and allo-SCT.

## Case presentation

**Fig. 1:**
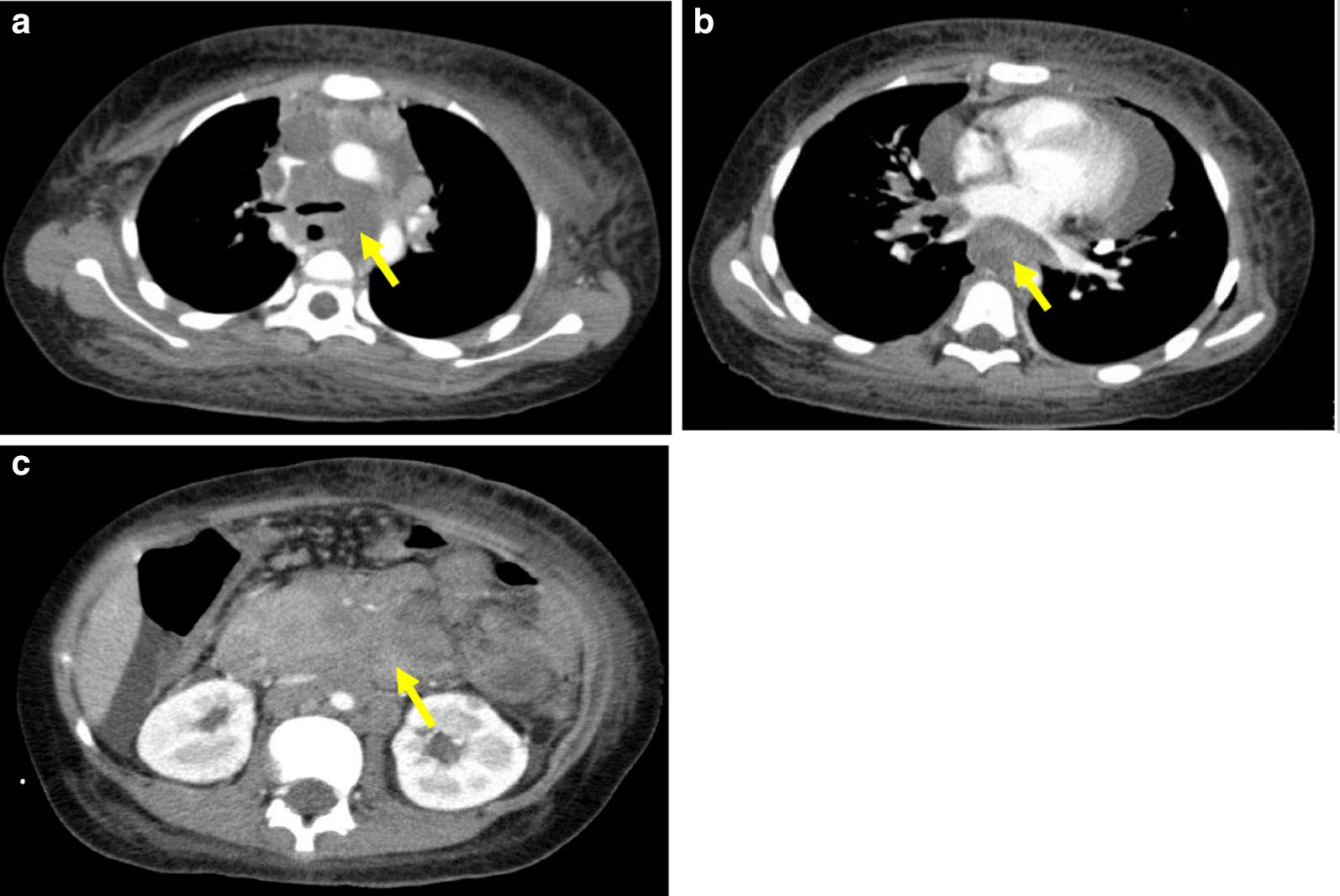
Contrast-enhanced computed tomography (CT) at admission. **a** Chest CT revealing mediastinal lymphadenopathy along with compression of the left and right bronchi (yellow arrow). **b** Chest CT revealing mediastinal lymphadenopathy along with compression of the esophagus (yellow arrow). **c** Abdominal CT revealing lymphadenopathy of the para-aorta, pancreas, and mesentery (yellow arrow)

An 11-year-old Japanese boy who complained of abdominal pain and cough presented to a local hospital in December 2016. Enhanced computed tomography (CT) revealed bilateral pleural effusion and marked lymphadenopathy in the mediastinum, epigastrium, mesentery, neck, and axillae on both sides, as well as around the thoracoabdominal aorta. These findings were indicative of an advanced malignant lymphoma (Fig. 1a–c). The patient was transferred to our hospital for further treatment 16 days after presentation. He was 142.0 cm in height and weighed 42.1 kg at the time of admission.

Laboratory examination findings at the time of admission to our hospital were as follows: total white blood cell count, 22,900/mm^3^; C-reactive protein level, 2.35 mg/dL; lactate dehydrogenase level, 607 IU/L; and soluble interleukin-2 receptor level, 203,887 U/mL.

**Fig. 2:**
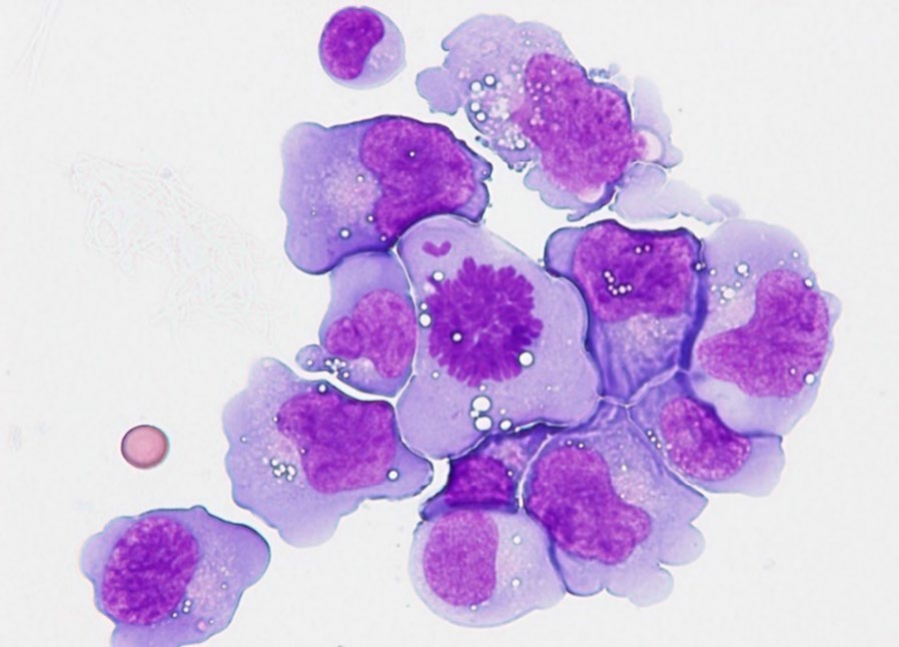
Pleural effusion cytology and flow cytometry findings. Pleural effusion specimens processed using the cytospin centrifuge revealing abnormally huge cells suspected to be malignant lymphoma cells

The pleural floating cell smear using cytospin (Fig. 2) and the bone marrow smear showed that 90 and 1.2% of the cells, respectively, were large abnormal cells with vacuoles. Flow cytometry revealed abnormal cells that were strongly positive for CD4, CD25, CD30, and CD45RO and partially positive for CD7. Moreover, the abnormal cells were positive for ALK on fluorescence in situ hybridization (15% in the pleural effusion sample and 2% in the bone marrow sample). The level of nucleophosmin-ALK mRNA in the bone marrow was elevated to 8765 copies/ABL 10,000 copies. Stage IV ALK-positive ALCL was diagnosed based on the above findings.

**Fig. 3:**
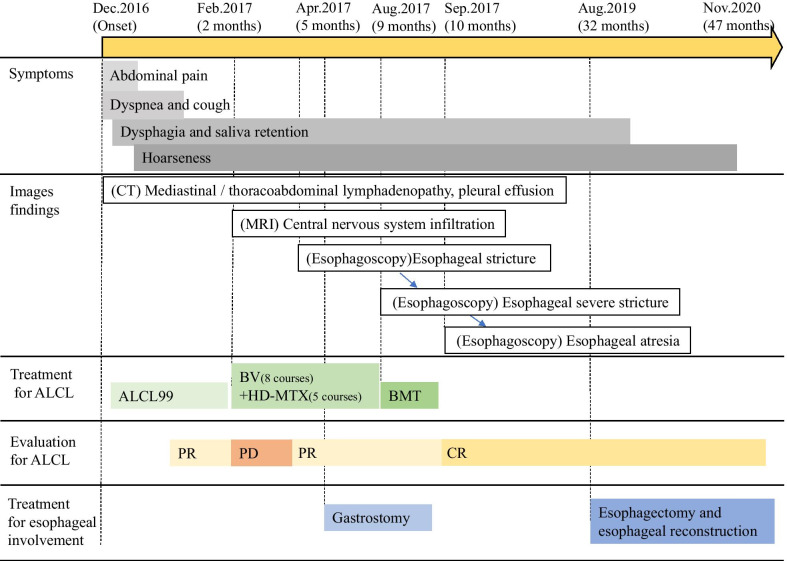
Clinical course. Clinical course and changes in symptoms, treatment, and images findings. *ALCL* anaplastic large cell lymphoma, *BV* brentuximab vedotin, *HD-MTX* high-dose methotrexate, *BMT* bone marrow transplantation, *PR* partial response, *PD* progressive disease, *CR* complete response

**Fig. 4:**
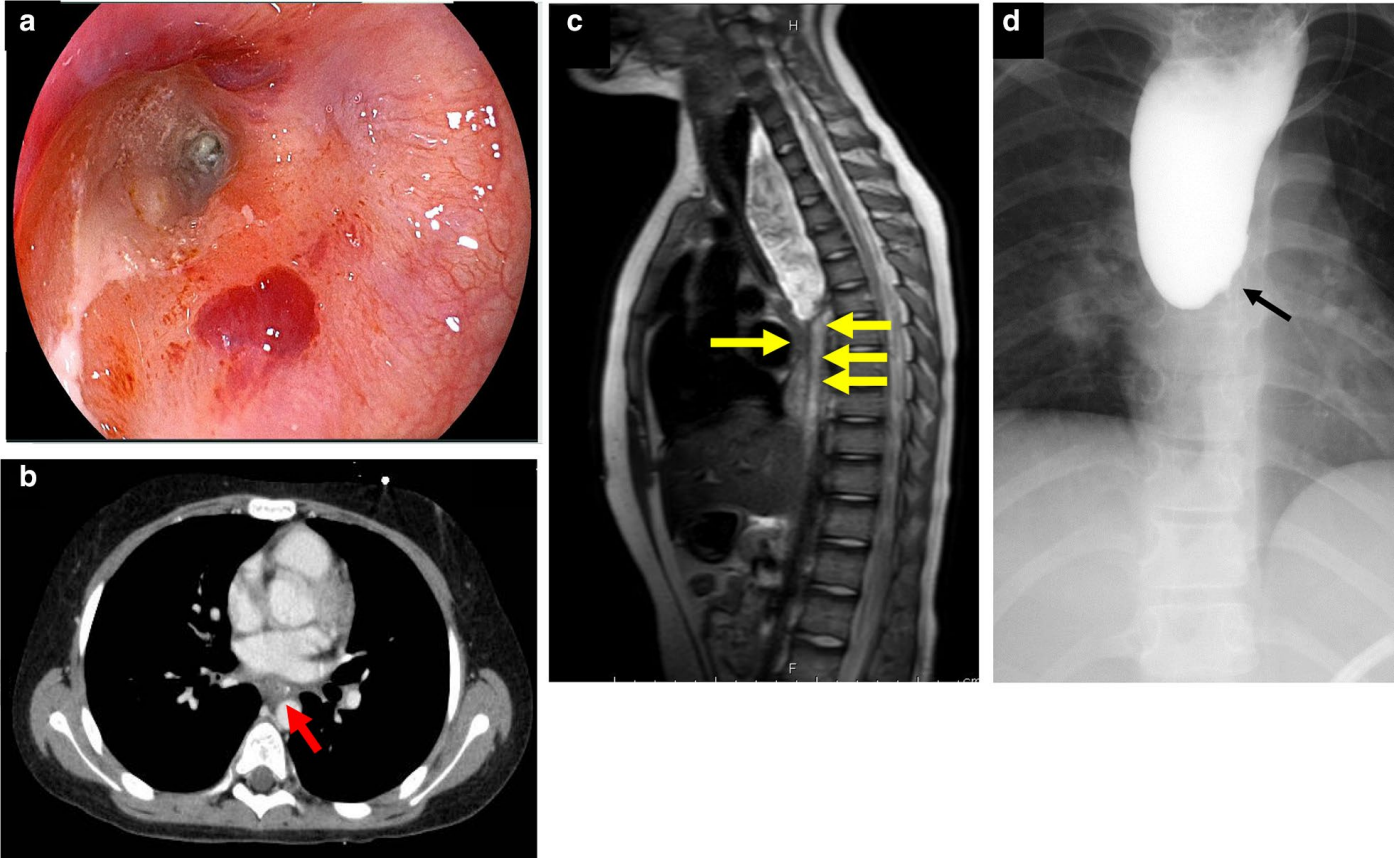
Imaging findings. **a** Esophagoscopy revealing a severe stricture along with circumferential wall thickening, which made the passage of the scope impossible. **b** Contrast-enhanced chest computed tomography during chemotherapy revealing reduced lymphadenopathy in the mediastinal lymph nodes (red arrow). **c** Contrast-enhanced magnetic resonance imaging during chemotherapy revealing the narrowing of the middle thoracic esophagus (yellow arrows). **d** Barium contrast imaging revealing a complete obstruction (black arrow) in the middle thoracic esophagus with proximal dilation

Nasogastric feeding was initiated to supplement nutrition and prevent aspiration owing to bilateral laryngeal nerve palsy. Chemotherapy with the ALCL 99 regimen was administered [5], resulting in temporary remission of the ALCL and improvement in the general condition of the patient; however, central nervous system infiltration was observed during the intermittent administration of chemotherapy (Fig. 3). Therefore, treatment with brentuximab vedotin (BV) and high-dose methotrexate (HD-MTX) was initiated. At 5 months after diagnosis, remission was observed; however, the patient complained of dysphagia and vomiting, and esophagoscopy revealed a severe stricture in the middle thoracic esophagus (Fig. 4a). Lymphadenopathy, including that in the mediastinum, was reduced on CT (Fig. 4b), and no obvious tumor was observed near the stricture site on magnetic resonance imaging (Fig. 4c). Esophageal mucosal biopsy of the stricture site also revealed no malignancy. A second esophagoscopy was performed 3 weeks after the first one; the findings revealed that the stricture had progressed to the pinhole in the part with the most severe stricture, and barium examination revealed a complete esophageal obstruction in the middle thoracic esophagus with proximal dilation (Fig. 4d). Gastrostomy was performed for feeding.

After 5 courses of HD-MTX and 8 courses of BV (i.e., 9 months after diagnosis), the patient underwent allogeneic bone marrow transplantation (allo-BMT) from a human leukocyte antigen-matched unrelated donor, one form of allo-SCT. The reduced conditioning regimen included fludarabine (30 mg/m^2^ × 5), melphalan (70 mg/m^2 ^×2), and total body irradiation (2 Gy × 2). Tacrolimus and short-term methotrexate were administered for acute graft versus host disease (GVHD). Tacrolimus was gradually decreased and discontinued 6 months after allo-BMT. Even after the discontinuation of tacrolimus, no complications such as chronic GVHD developed.

**Fig. 5:**
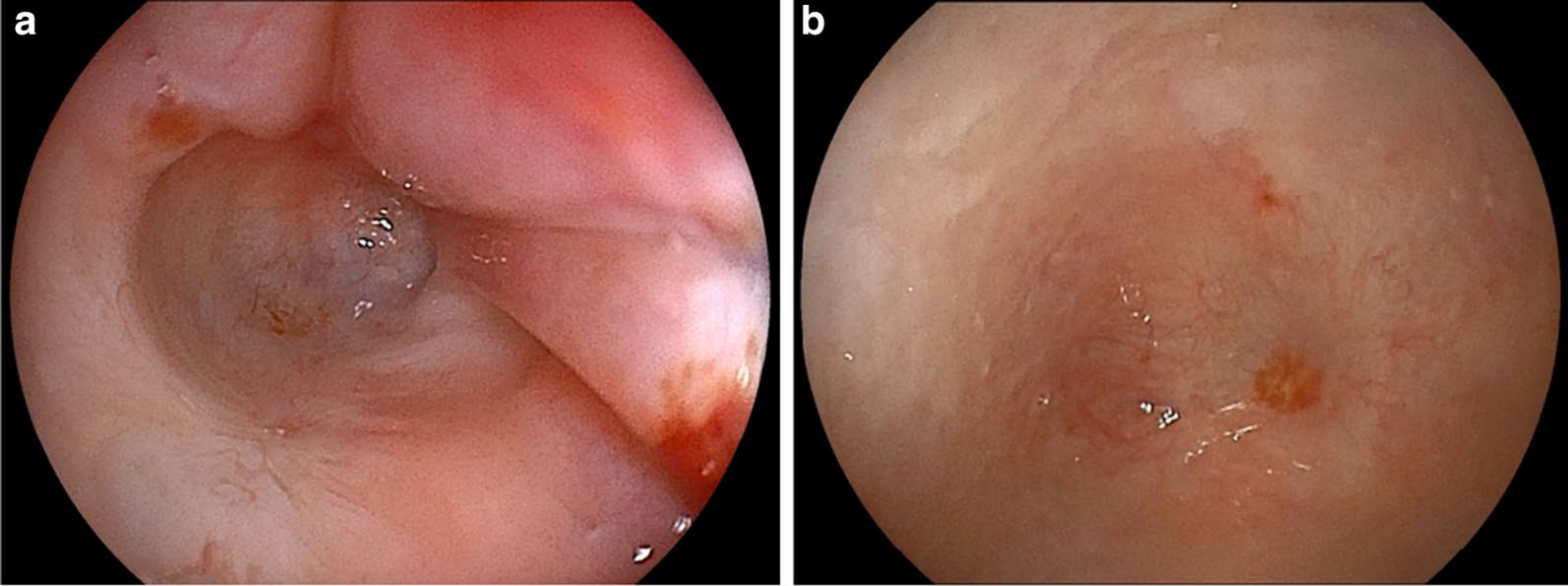
Esophagoscopy findings. **a** Esophagoscopy revealing a blind end—26 cm from the incisors—in the middle thoracic esophagus, similar to that in congenital esophageal atresia, at approximately 10 months after diagnosis. **b** Esophagoscopy via gastrostomy revealing a blind end in the lower thoracic esophagus, at approximately 2 years after diagnosis

After allo-BMT, complete remission continued to be maintained, and the patient was discharged; however, dysphagia and saliva retention did not improve. At approximately 10 months after diagnosis, esophagoscopy revealed a blind end—26 cm from the incisors—in the middle thoracic esophagus, similar to that observed in congenital esophageal atresia (Fig. 5a). Esophageal reconstruction was necessary for maintaining oral intake, and to wait for recovery from immunosuppression, we decided to perform esophagectomy more than 2 years after allo-BMT.

**Fig. 6:**
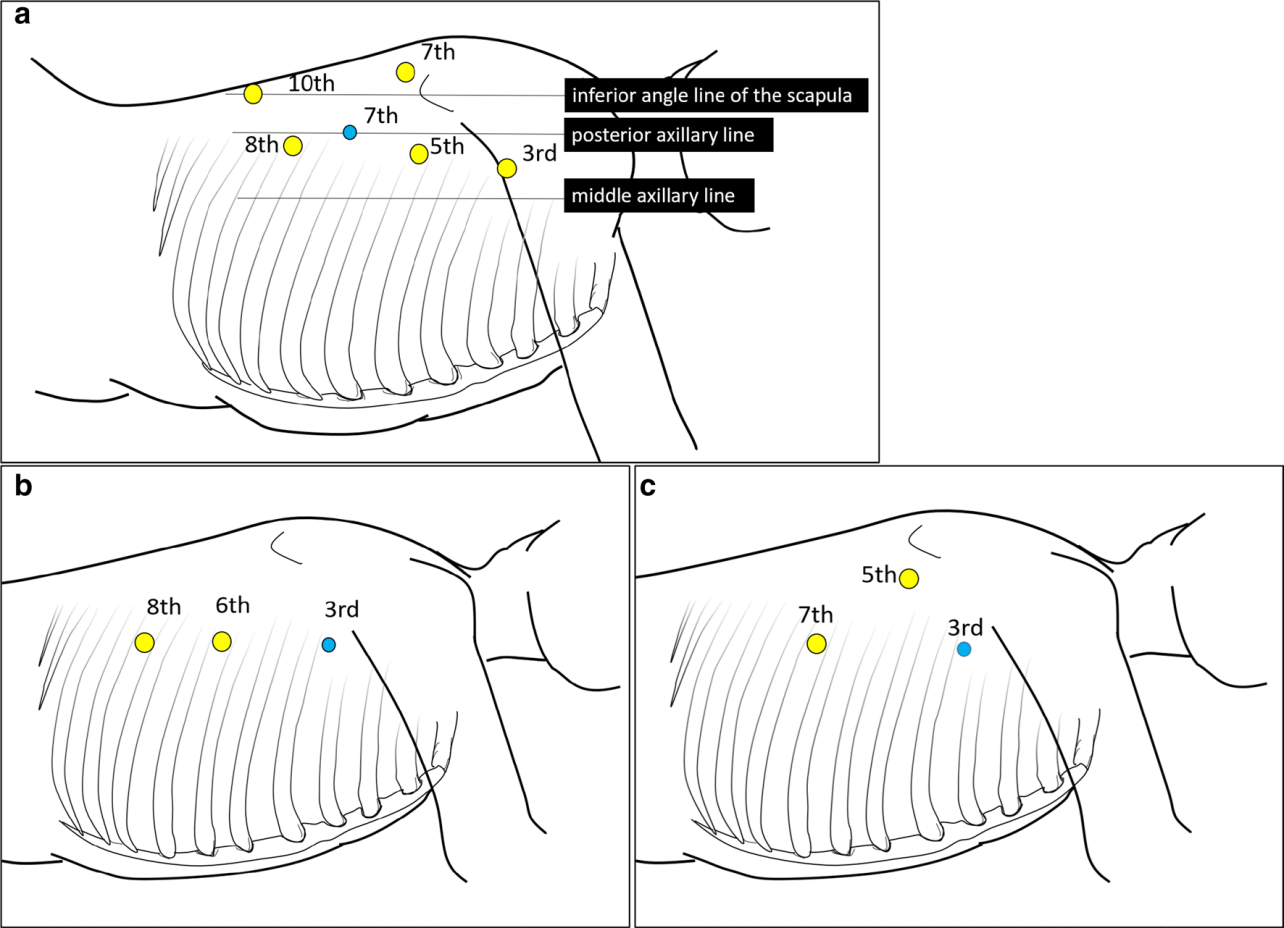
Port arrangements. **a** Port placement in this case. Six ports were placed thoracoscopically. The yellow circle indicates the 5-mm port, and the blue circle indicates the 3-mm port. **b** Port placement in a pediatric patient with type A congenital esophageal atresia at our hospital. Three ports are placed thoracoscopically: one each at the 3rd, 6th, and 8th intercostal spaces at the posterior axillary line. **c** Port placement in a pediatric patient with type C congenital esophageal atresia at our hospital. Three ports are placed thoracoscopically: one at the 3rd intercostal space at the posterior axillary line, one at the 7^th^ intercostal space at the posterior axillary line, and one near the tip of the scapula

Approximately 2 years after diagnosis, esophagoscopy was performed again with findings similar to those obtained 1 year and 2 months previously. Esophagoscopy via gastrostomy revealed a blind end in the lower thoracic esophagus (Fig. 5b). At 2 years and 8 months after diagnosis, the patient was readmitted for esophageal reconstruction. He was 153.5 cm in height and weighed 52.4 kg at the time of operation. Laboratory examinations showed normal findings: white blood cell count, 7040/mm^3^; neutrophil count, 2640/mm^3^; lymphocyte count, 3650/mm^3^; serum albumin level, 4.4 g/dL; serum IgG level, 1752 mg/dL; CD4 T-cells, 33% of the total lymphocytes; CD8 T-cells, 40% of the total lymphocytes; CD4/CD8 ratio, 0.83 (normal range: 0.8–3.5); and proliferation in response to phytohemagglutinin stimulation, 36,280 cpm (normal range: 34,400–62,300 cpm). His immune function was restored to the normal state with no onset of chronic GVHD. His performance status score was 0, and his physical condition and nutritional status were good except for hoarseness and saliva retention. He underwent a minimally invasive subtotal esophagectomy under thoracoscopy and laparoscopy. First, the patient was placed in the prone position during thoracoscopy. We placed 5 and 3 mm thoracoscopic ports as shown in Fig. 6a and maintained the intrathoracic pressure at a positive pressure of 5 mmHg under two-lung ventilation. Fibrinous adhesions were observed across the entire mediastinum. We proceeded with dissection along the esophagus. The esophagus was significantly adhered to the aorta, trachea, and pericardium but was detachable. We did not perform lymph node dissection. Second, we placed the patient in the supine position and performed hand-assisted laparoscopic mobilization of the stomach. We removed the granulation tissue of the fistula that had formed in the anterior wall of the antrum of the stomach after the gastrostomy and sutured the fistula shut. We created a gastric conduit at the greater curve via the retrosternal route and performed a cervical esophagogastric anastomosis. The total operation time was 548 min, and the total amount of intraoperative bleeding was 80 mL.

**Fig. 7:**
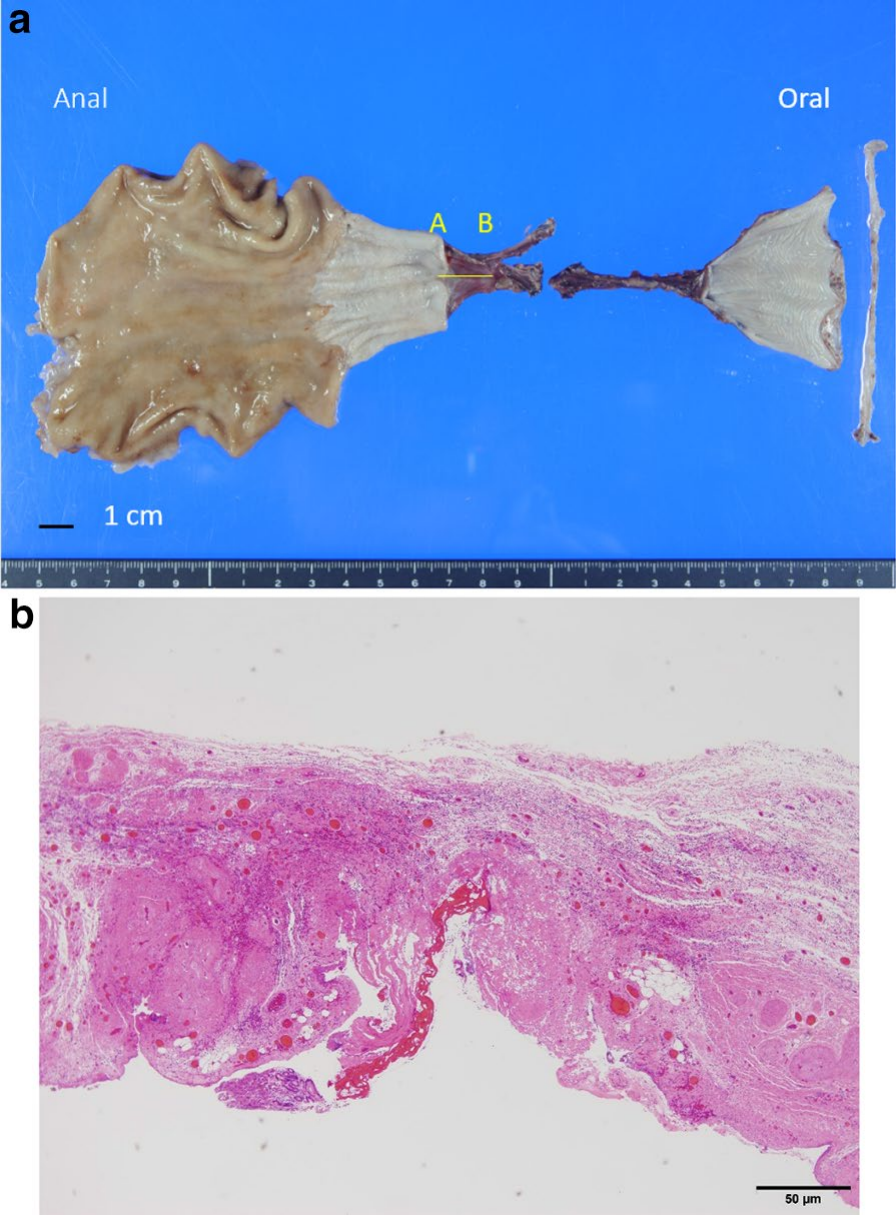
Histopathological findings. **a** Gross findings of the resected specimen revealing the disruption of the esophageal epithelium for approximately 75 mm in the middle thoracic esophagus. **b** Microscopic examination of the area denoted with the yellow line (from **a** to **b**) in Fig. 6a (hematoxylin and eosin staining; magnification, × 100) revealing no esophageal structures and replacement of esophageal epithelium by hyalinosis and fibrosis

Pathological examination of the surgical specimens revealed no esophageal epithelium, which had been replaced by hyalinosis and fibrosis (Fig. 7a, b). No viable cells were identified in the entire esophagus, stomach, and lymph nodes.

The patient did not experience any major postoperative complications, although he did complain of dysphagia for a while after the operation. As there was no problem with the passage on barium contrast imaging, the patient started receiving swallowing training. He was transferred to the local hospital for swallowing training on postoperative day 68. Subsequently, the symptoms of dysphagia disappeared, food could pass easily, and complete remission was still maintained at 14 month post-surgery.

## Conclusions and discussion

Extranodal sites, including the subcutaneous tissue, bones, bone marrow, and spleen, have been associated with ALCL, although they have rarely been associated with the gastrointestinal tract [2, 3]. Moreover, esophageal involvement of ALCL is even rarer [4]. The optimal timing of treatment and the post-onset course for esophageal involvement of ALCL are unclear. In addition, there are few reports of cases of esophagectomy after allogeneic hematopoietic stem cell transplantation (allo-SCT). Thus, we performed a search of studies published in English literature about ALCL with esophageal involvement, and esophagectomy after allo-SCT, respectively.

A total of 8 cases of ALCL with esophageal involvement—including the current case—have been reported (Table 1) [4, 6–11]. Of the 8 cases, 5 were diagnosed as primary esophageal lymphoma, and in 3 cases, including the current case, patients showed the esophageal involvement in systemic ALCL. Six of the 8 cases were ALK-positive ALCL. All the patients were treated with chemotherapy, but in 1 case, in which the patient was preoperatively diagnosed with an esophageal submucosal tumor, esophagectomy was performed for treatment. The complications of esophageal involvement included esophageal stricture in 3 cases, esophageal obstruction and perforation in 1, esophageal dilation owing to the tumor in 1, and tracheoesophageal fistula in 1. The current case is the only case of progression to esophageal cicatricial atresia.

**Table 1:** Summary of clinical features of anaplastic large-cell lymphoma cases with esophageal involvement

	Author (reference number), year	Age (years)	Sex	Symptoms and signs	Esophageal site of the tumor	Tumor type (macroscopic appearance on endoscopy)	Complication owing to esophageal involvement	Diagnostic method	Anaplastic lymphoma kinase (ALK) expression	Tumor stage per the Ann arbor classification	Treatment	Outcome
1	Pearson JM et al. [6], 1991	73	M	Fatigue, weight loss, fever	Lower thoracic	Infiltrative type	Esophageal dilation	Autopsy	Not described	IV	None	Death immediately after onset
2	Ross CW et al. [7], 1992	44	F	Dysphagia, back pain	Upper thoracic	Protruding type	Severe esophageal stricture	Endoscopic biopsy of the esophagus	Not described	IV	Chemotherapy and radiotherapy	Lost to follow-up after 6 months
3	Yaakup H et al. [8], 2008	34	F	Dysphagia	Upper thoracic	Protruding type (similar to hard fungating tumors)	Severe esophageal stricture	Endoscopic biopsy of the esophagus	Positive	IV	Chemotherapy, allo-SCT, and esophageal stenting	Being followed up for 24 months
4	Joshi A et al. [9], 2008	55	M	Dysphasia, weight loss, sore throat	Cervical	Ulcerative type	Tracheoesophageal fistula	Endoscopic biopsy of the esophagus	Positive	III	Chemotherapy and allo-SCT	Not described
5	Wu N et al. [4], 2011	37	M	Dysphagia, weight loss	Middle thoracic	Protruding type (submucosal tumor)	None	Esophagogastrostomy	Positive	III	Esophagectomy and adjuvant chemotherapy	Being followed up for 14 months
6	Hryhorczuk AL et al. [10], 2012	3	M	Abdominal pain, vomiting	Upper thoracic	Not described	Esophageal obstruction and perforation	Axillary lymph node biopsy	Positive	III	Chemotherapy	Not described
7	Azarpira N et al. [11], 2017	16	M	Dysphagia	Middle thoracic	Infiltrative type	Severe esophageal stricture	Endoscopic biopsy of the esophagus	Positive	III	Chemotherapy and esophageal dilatation	Not described
8	Current case	11	M	Abdominal pain, cough, dysphagia	Middle thoracic	Infiltrative type	Esophageal atresia	Chest effusion cytology	Positive	IV	Chemotherapy, allo-SCT, and esophagectomy	Being followed up for 47 months

*allo-SCT* allogeneic hematopoietic stem cell transplantation, *ALK* anaplastic lymphoma kinase

Although there have been no previous reports on the esophageal involvement of lymphoma that resulted in esophageal cicatricial atresia similar to congenital esophageal atresia, acquired intestinal atresia after necrotizing colitis or adhesion has been frequently reported in the gastrointestinal tract [12–14]. And if inflammation occurs, acquired atresia might occur during the healing process. Among previously reported cases, some patients showed progression from esophagitis and mucosal damage to the esophageal stricture owing to chemotherapy, radiotherapy, or *Candida* infection [14–16]. In the current case, chemotherapy might have been an influencing factor; however, the most significant cause of acquired esophageal atresia appeared to be esophageal infiltration or desmoplastic reactions associated with extranodal infiltration from the mediastinal lymph nodes. This might have occurred owing to a series of mechanisms that caused the stricture: from infiltration to progression to complete occlusion owing to desmoplastic reactions [9]. The occluded portion of the esophagus structure was replaced by fibrous tissue; only the epithelium was covered during the healing process, finally resulting in a blind end. These changes occurred during the course of chemotherapy.

The standard treatment for ALCL is chemotherapy [3]. However, when patients with ALCL have the complications of esophageal obstruction, hemorrhage, or perforation, surgical intervention may be required [6]. Yaakup et al. reported that esophageal dilatation and esophageal stent placement could improve the symptoms of dysphagia for esophageal stricture with ALCL, which may be an effective treatment option [8]. In the current case, we were unaware that the esophageal stricture would eventually lead to esophageal cicatricial atresia and could not perform stent placement and dilation for the esophageal stricture. Therefore, esophageal reconstruction was the necessary approach. The length of the gap is an important factor when considering the method of reconstruction in patients with esophageal atresia. Reconstructive treatment options for congenital long-gap esophageal atresia (LGEA) include delayed primary anastomosis, esophageal myotomy, extrathoracic esophageal elongation (Kimura technique), internal traction-induced esophageal growth (Foker process), and thoracoscopic repair. The choice of method depends largely on the gap length, any associated anomalies, and the size and quality of the esophageal ends [17]. Esophageal replacement may be required in cases of LGEA complicated by anatomical leaks, refractory stricture, and recurrence of tracheoesophageal fistula. However, the current case involved esophageal atresia caused by extraesophageal infiltration of lymphoma, and the gap was also long. Therefore, one-stage esophageal replacement was selected. Various conduits, such as the stomach, colon, and small bowel, are currently used for esophageal replacement worldwide; however, each method has its own problems and complications, and there is little consensus on the superior method in terms of mortality, postoperative morbidity, and long-term functional outcomes [17–20].Long-term complications of gastroesophageal reflux, stricture formation, dumping syndrome, and dysphagia may occur with esophageal reconstruction using gastric conduits. The use of the small intestine and colon is expected to be associated with lower rates of these complications, but unfortunately there is little consensus. A gastric conduit has abundant blood flow and long-term patency [21, 22]. Additionally, esophageal reconstruction using a gastric conduit is the most frequently performed approach in our department Thus, a gastric conduit was selected in this case. We also selected the retrosternal route in consideration of the cosmetic outcome and the possibility of recurrence in the mediastinum.

Minimally invasive esophagectomy using a thoracoscope or laparoscope has been performed for pediatric cases [23–25]. Each institution has its preferred port arrangement for thoracoscopic esophagectomy. At our hospital, the patient is placed in the left semi-prone position. The port arrangement shown in Fig. 6b is used for infants and toddlers with type A congenital esophageal atresia and that shown in Fig. 6c is used for those with type C congenital esophageal atresia. In the current case, the patient was 13 years at the time of the operation, and his physique was not significantly different from that of an adult. Therefore, we performed minimally invasive esophagectomy combined with a thoracoscopic and hand-assisted laparoscopic surgery and gastric conduit reconstruction, as well as esophagectomy, as performed for adult patients with esophageal cancer. Fortunately, no issues were encountered when fashioning the gastric conduit; however, use of a gastrostomy meant that a longer surgical time than usual was required. As severe esophageal stricture may progress to esophageal atresia, assuming that esophagectomy and gastric conduit reconstruction will be performed later, it may be better to perform jejunostomy instead of gastrostomy for establishing a feeding route.

To the best of our knowledge, no previous study has determined the optimal time to perform esophagectomy after allo-SCT. However, it is desirable to avoid major surgeries such as esophagectomy immediately after allo-SCT, because it is immunocompromised, and immunity after allo-BMT improves slowly after 1–2 years if the patients have no chronic GVHD after the discontinuation of immunosuppressive drugs [26, 27]. As the current case did not have chronic GVHD and the immunosuppressive drugs could be discontinued early, esophagectomy was performed 2 years after the improvement of immunity was expected, and esophagectomy was possible without major problems. However, Kato et al. [28] reported the use of esophagectomy for secondary esophageal cancer that occurred after allo-SCT in 10 cases; they observed postoperative pneumonia in 5 cases even after an average of 9 years 8 months (range: 4 years 8 months–14 years 8 months). Therefore, it is necessary to consider the possibility of infection and be ready to manage it, because patients may be immunosuppressed even after a long time following allo-SCT.

In conclusion, oncologists must remain alert for passage disturbance owing to acquired esophageal cicatricial atresia that can occur as a complication during the course of chemotherapy for ALCL with esophageal infiltration. If the unlikely event of esophageal obstruction or esophageal atresia occurs and if remission is maintained, esophagectomy and esophageal reconstruction are useful treatment methods for oral intake. Additionally, although it is necessary to consider the possibility of infection even after a long time following allo-SCT, esophagectomy may be successful if the patient does not have.

## Data Availability

The datasets supporting the conclusions of this article are included within the article and its additional files.

## Abbreviations

ALCL: Anaplastic large cell lymphoma
ALK: Anaplastic lymphoma kinase
allo-SCT: Allogeneic hematopoietic stem cell transplantation
CT: Computed tomography
BV: Brentuximab vedotin
HD-MTX: High-dose methotrexate
allo-BMT: Allogeneic bone marrow transplantation
GVHD: Graft versus host disease
LGEA: Long-gap esophageal atresia
